# Extracts of *Lycoris aurea* Induce Apoptosis in Murine Sarcoma S180 Cells

**DOI:** 10.3390/molecules17043723

**Published:** 2012-03-26

**Authors:** Na Liao, Mingzhang Ao, Peng Zhang, Longjiang Yu

**Affiliations:** Department of Biotechnology, Institute of Resource Biology and Biotechnology, College of Life Science and Technology, Huazhong University of Science and Technology, Wuhan 430074, Hubei, China; Email: amz25@163.com (M.A.); zhangpenghust@126.com (P.Z.)

**Keywords:** *Lycoris aurea*, anticancer activity, apoptosis, lycorerine-type alkaloid

## Abstract

*Lycoris* species have been known since long ago as a multi-utility ethnomedicinal herbal in China. It has been reported to exhibit a number of properties such as anticancer, neuroprotective, and antibacterial activities. In the present study, the anticancer efficacy of dichloromethane extracts of *Lycoris aurea* (DELA), was evaluated both *in vivo* and *in vitro* using murine sarcoma 180 cells. To evaluate the effects of DELA on apoptotic cell death, flow cytometry and Western blotting were performed. DELA demonstrated promising inhibition effects on sarcoma 180 cells *in vitro* and a 53.49% inhibitory rate on cancer cells *in vivo*. DELA treatment increased thymus indices and spleen indices *in vivo*, indicating that it reduced tumours, but did not damage the main immune organs. The DELA-evoked increase in apoptotic cell death was accompanied by occurrence of cleaved caspase-3 and decreases in the ratio of Bcl-2/Bax. Further purification and LCMS analysis showed DELA contained homolycorine, 2α-hydroxyoduline, oduline, hippeastrine, 2α-hydroxy-6-*O*- methyloduline, and 2α-methoxy-6-*O*-methyloduline. These results indicate that DELA exerted its anticancer effects, at least in part, by inducing cancer cell apoptosis and thus can be considered as a potential candidate agent for treatment of cancer.

## 1. Introduction

Cancer is a disease intimately connected with apoptosis. Apoptosis is both a form of cell death and a complex endogenous gene-controlled event [1]. An abnormality in apoptosis causes malignancy, which may lead to uncontrolled cell growth. Therefore, the induction of cancer cell apoptosis is a very useful method of cancer therapy. However, the efficacy of anticancer drugs with apoptotic effects, such as cyclophosphamide (CTX) [2], are limited by a range of confounding factors including nausea, vomiting, and immunotoxicity [3]. Therefore, exploring agents that can effectively induce cancer cells apoptosis with low immunotoxicity are required.

*Lycoris aurea* (*L. aurea*) is a species of Amaryllidaceae plant distributed in China. Strong basic alkaloids extracts of *L. aurea* such as lycorine and galanthamine show anticancer, neuroprotective, and antibacterial activities [4]. Lycorine has been demonstrate to inhibit human leukemia cell line HL-60 and human leukemia cell line HL-60 growth by arresting the cell cycle, and it mediated cancer cell apoptosis by the cytochrome c-mediated and caspase-dependent pathway [5,6,7]. However, no systematic studies on the anticancer activity of weak basic alkaloids extracts of *L. aurea* have been published to date. 

In the present study, dichloromethane extract of *Lycoris aurea* (DELA) was prepared, further purified and analyzed by LCMS. The antitumour activity of DELA was tested on murine sarcoma S180 cells *in vivo* and *in vitro*. Annexin V-FITC/propidium iodide (PI) double-staining assays were employed to detect DELA-induced murine sarcoma S180 cell apoptosis, and Western bloting analysis was employed to investigate the expression of cell death regulator molecules like Bax, Bcl-2, and cleaved caspase-3.

## 2. Results and Discussion

### 2.1. Antitumour Activity *in Vitro* and *in Vivo*

**Figure 1:**
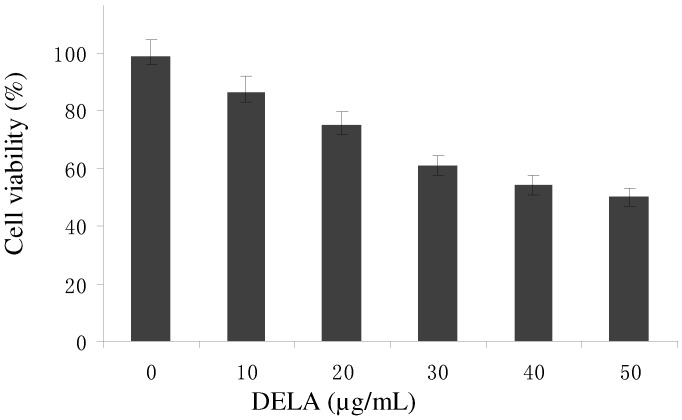
Comparison of the inhibitory effects of DELA on murine sarcoma S180 cells. Data are shown as mean ± SD (n = 3).

DELA exerted significant inhibitory effects on murine sarcoma S180 cells in a dose-response manner (Figure 1). Treatment with the three DELA doses resulted in marked suppression of tumour weight (Table 1). Compared with the control model, the inhibitory rates of DELA at low, middle, and high doses were 27.9%, 37.21%, and 53.49%, while cyclophosphamide (CTX) caused 68.22% inhibition. Thus, the high DELA dosage group showed significant reduction of tumour weight compared with the control model and the inhibitory rate is significantly higher than the 39.82% antitumour inhibitory rate of one potential anticancer agent [8], and closer to the 53.01% efficacy shown by another potential anticancer agent wogonin [9]. 

After drug administration, the thymus indices of high DELA dosage groups were significantly higher than both the control group and the CTX group. The spleen indices of the DELA sample groups were also significantly higher than both the control group and the CTX group (Table 1). Thymus and spleen are the main immune organs and are responsible for initiating immune reactions in the body [10]. The indices of thymus and spleen directly reflect the status of the immune system. Increases in these indices [11] suggest that DELA did not cause any serious toxic effects on the immune system. In contrast, CTX exhibited serious toxic effects on the immune system of mice, and possesses a number of previously reported side effects [12], but is still used in the clinic. Further studies have to show whether DELA has less side effects.

**Table 1:** Effects of DELA on S180 in Mice (Mean ± S.D.) (*n* = 10).

Group	Dose (mg/kg)	Weight (g)		Tumor weight (g)	Inhibitory rate (%)	Thymus index (mg/g)	Spleen index (mg/g)
		Before treatment	After treatment				
model control	-	19.49 ± 1.21	28.36 ± 3.05	1.29 ± 0.38		2.19 ± 0.80	7.43 ± 1.29
CTX	20	19.33 ± 1.38	22.57 ± 1.47	0.41 ± 0.12 **	68.22	1.03 ± 0.16	5.28 ± 1.04
DELA	120	19.18 ± 1.57	27.94 ± 2.25	0.60 ± 0.14 **	53.49	2.98 ± 0.61 *^a^	9.95 ± 1.87 *^a^
DELA	40	19.44 ± 1.82	28.35 ± 2.61	0.81 ± 0.19	37.21	2.46 ± 0.53 ^a^	9.22 ± 1.59 ^a^
DELA	20	19.61 ± 1.16	29.17 ± 2.79	0.93 ± 0.22	27.9	2.23 ± 0.59 ^a^	8.98 ± 1.40 ^a^

CTX is the abbreviation of cyclophosphamide. ^a^ Significantly different from CTX group at *p* < 0.01; * significantly different from model control group at *p* < 0.05; ** significantly different from model control group at *p* < 0.01.

### 2.2. DELA-Induced Apoptosis in Murine Sarcoma S180 Cells

**Figure 2:**
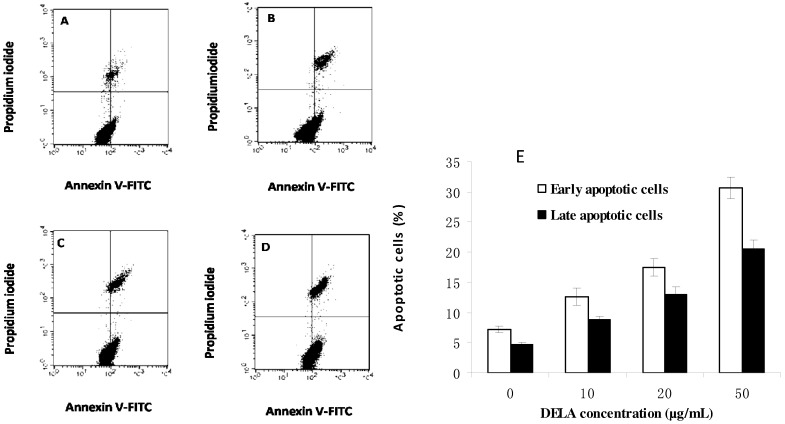
Annexin V/PI double-staining assay of murine sarcoma S180 cells treated with (**A**) 0, (**B**) 10, (**C**) 20, and (**D**) 50 µg/mL DELA. The Y-axis represents the PI-labelled population, whereas the X-axis represents the FITC-labelled Annexin V positive cells. The lower left portion of the fluorocytogram (An−, PI−) shows normal cells, whereas the lower right portion of the fluorocytogram (An+, PI−) shows early apoptotic cells. The upper right portion of the fluorocytogram (An+, PI+) shows late apoptotic cells. (**E**) The apoptotic rates of murine sarcoma S180 cells induced by DELA.

Early apoptosis is characterized by the translocation of phosphatidylserine (PS) from the inner layer of the plasma membrane to the outer surface [13]. Quantitation of Annexin V-FITC binding to externalized PS can quantitate apoptosis cells. After treatment with 0, 10, 20, and 50 µg/mL DELA for 24 h, the early apoptotic rates were 7.17%, 12.64%, 17.52%, and 30.71%, respectively, and the late apoptotic rates were 4.64%, 10.76%, 12.99%, and 20.54% (Figure 2). These results indicate that DELA-induced apoptosis in murine S180 cells.

### 2.3. DELA-Induced Procaspase-3 Protein Cleaved in Murine Sarcoma S180 Cell

**Figure 3:**
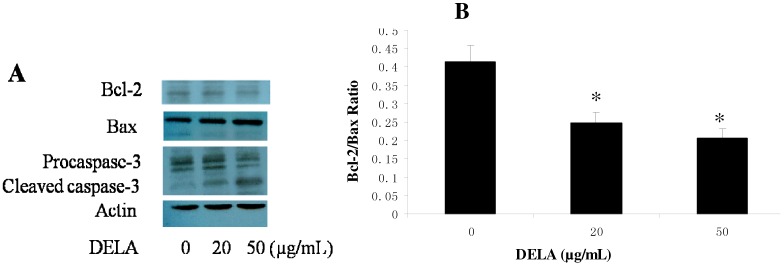
Western blotting analysis of Bax, Bcl-2, and procaspase-3 of the murine sarcoma S180 cells treated with 0, 20, and 50 µg/mL of DELA for 12 h. (**A**) Levels of Bcl-2, Bax, procaspase-3, and cleaved caspase-3, (**B**) Graphs showing changes in the ratio of Bcl-2/Bax in cells. The results are expressed as means ± SD (n = 3). * *p* < 0.01, compared with 0 µg/mL.

The effects of DELA on levels of Bcl-2 family proteins and caspase-3 were also examined by Western blot analysis. After murine sarcoma S180 cells were treated with DELA for 12 h, the expression of Bcl-2 protein decreased, while the expression of the pro-apoptotic Bax protein increased compared to the control, resulted in decreasing the ratio of Bcl-2/Bax (Figure 3B). With the decreased expression of procaspase-3 protein, cleaved caspase-3 occurred (Figure 3A).

Apoptosis is a normal component of the development and health of multicellular organisms and a cell-intrinsic programmed suicide mechanism that results in the controlled breakdown of the cell into apoptotic bodies. Caspases (cysteine-aspartic acid proteases) are crucial mediators of apoptosis. Among them, caspase-3 is a frequently activated death protease, catalyzing the specific cleavage of many key cellular proteins [14]. Activation of the effector caspase-3 was assessed using an antibody that recognizes full-length inactive and uncleaved procaspase-3. Decrease in the level of uncleaved procaspase-3 accompanys with increasing in cleaved caspase-3. Cleaved caspase-3 occurred is considered a sign of caspase-3 activation. And active caspase-3 is a marker for cells undergoing apoptosis [15,16]. These results indicate that DELA-induced apoptosis in murine sarcoma S180 cells. 

The Bcl-2 protein was the first proto-oncogene found with anti-apoptotic functions and is overexpressed in breast cancer, prostate cancer, and colorectal adenocarcinoma [17]. The anti-apoptotic Bcl-2 has generated significant attention as a potential apoptosis regulator in cancer treatment [18] since reports demonstrating that antisense RNA induced leukaemic cell apoptosis by suppression of Bcl-2. Consequently, down-regulation of Bcl-2 is another desired property of anticancer drugs. However, Bcl-2 family proteins include both anti-apoptotic members and pro-apoptotic members, including the Bax protein. A decreased ratio can activate the mitochondria apoptotic cell death pathway [19,20]. In the present study, DELA decreased the ratio of Bcl-2/Bax, which suggests that the mitochondrial apoptotic pathway was activated by DELA during murine S180 cell apoptosis.

### 2.4. Structural Elucidation of Isolated Compound

The NMR spectra of the first compound isolated from DELA were identified as those of homolycorine. The MS characteristic fragmentations of the lycorenine-type alkaloids are seen at *m/z* 125 and 96, and originate from a retro-Diels-Alder cleavage of ring C, with a hydroxyl group in position 2. Other ions such as *m/z* 190, 162, 134, and 133 are significant because they convey important structural information. For example, two ion peaks of hippeastrine at *m/z* 125 (C and D ring) and 190 (A and B ring) completely account for the two parts of the molecule (*i.e.*, 125 + 190 = 315 = M^+^) [21]. The molecular ions and the mass fragments are listed in Table 2. Peak **4** contains 191, 126, and 96 ions. In the positive ion mode, the most prominent ion corresponded to [M+H]^+^. 191 and 126 were [M+H]^+^ ions in peak **4**. Other ions in peak 4 were identified as those of hippeastrine [22].

**Table 2:** Alkaloids in the extract of *Lycoris aurea*.

Peak No.	Identification	t _R_ (min)	[M+H]^+^	MS^2^ Characteristic ions (*m/z*)
**2**	2α-Hydroxyoduline	3.2	318	318, 282, 253, 193
**3**	Oduline [23]	4.4	302	302, 284, 256, 226, 193, 177, 145, 110, 94
**4**	Hippeastrine [22]	7.8	316	316, 298, 280, 239, 223, 191, 126, 96
**5**	2α-Hydroxy-6-*O*-methyloduline [22]	9.2	332	332, 314, 300, 282, 257, 239, 223, 201, 175, 96
**6**	2α-Methoxy-6-*O*-methyloduline	16.9	346	346, 314, 282, 264, 239, 199, 175

The M^+^ and characteristic ions of peak **3** and **5** were identified as those of oduline and 2α-hydroxy-6-*O*-methyloduline, respectively [22,23]. The characteristic ions of peak 2 were similar to peak **3** at 193, just more 16 mass numbers in the C and D rings. This indicated peak **2** was 2α-hydroxyoduline. The characteristic ions of peak 6 were similar to peak **5** at 175, just more 14 mass numbers in the C and D ring. This indicated peak **6** was 2α-methoxy-6-*O*-methyloduline.

**Figure 4:**
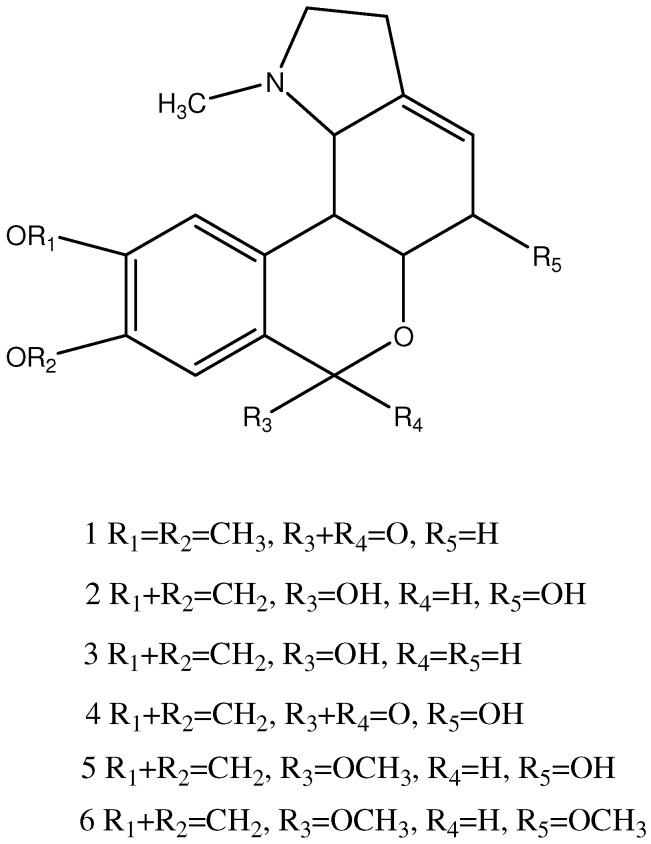
Structure of compounds in *L. aurea*.

In order to explore cancer cell apoptosis agents with low immunotoxicity, we evaluated DELA both *in vivo* and *in vitro* using murine sarcoma 180 cells. The results indicated that it reduced tumours by inducing cancer cells apoptosis but did not damage the main immune organs. Further purification and LCMS found DELA contained a series of lycorenine-type alkaloids such as homolycorine, 2α-hydroxyoduline, oduline, hippeastrine, 2α-hydroxy-6-*O*-methyloduline, and 2α-methoxy-6-*O*- methyloduline (Figure 4). 

Amaryllidaceae plants are widely distributed in several countries, and extensively used in traditional medicine. They are also cultivated as ornamental plants for their beautiful ﬂowers and for the production of volatile oil. Some Amaryllidaceae plants like had been used to treated cancer in the middle ages in China, North African, Central American, and Arabian medicine [24,25]. Their biological activities are frequently associated with several typical alkaloids they synthesize. Lycorine, which shows antitumor and antiviral activities, was the first alkaloid isolated from Amaryllidaceae species in 1877 [26]. To date, more than 100 alkaloids possessing a wide spectrum of biological activities have been isolated from various Amaryllidaceae species. They were often divided into several types based on their skeleton such as lycorine-type, galanthamine-type, lycorerine-type, tazettine-type, crinine-type, and narciclasine-type [27]. In some references, crinine-type also called haemanthamine-type. Homolycorine and its derivatives were divided into homolycorine type [28] and were not lycorerine-type. Lycorine and narciclasine show significant anticancer activies. The antitumor mechanism of lycorine was that it inhibited protein synthesis and cell apoptosis of MM46 in the presence of calprotectin [29], and suppressed leukemia growth and reduced cell survival via arresting cell cycle and inducing apoptosis [5]. Narciclasine’s anticancer mechanism was antimitotic [30]. Carrasco *et al*. [31] reported that narciclasine interacts with the 60S eukaryotic ribosome subunit and inhibits the peptide bond formation step in eukaryotic protein synthesis. More recently, Dumont *et al*. reported that narciclasine at concentrations of 1 µM appears to induce marked apoptosis-mediated cytotoxic effects in human carcinoma cells but not in normal ﬁbroblasts by activation of the death receptor pathway [32]. Pancratistatin was ﬁrst isolated from the tropical spider lily *Hymenocallis littoralis* in 1984 [33]. Pancratistatin treatment causes phosphatidyl-serine flipping, caspase-3 activation, generation of reactive oxygen species (ROS), and loss of mitochondrial membrane potential, which leads to apoptosis in cultured T-cell (Jurkat) leukemia cells [34]. McNulty *et al*. [35] reported that crinamine and haemanthamine, that belonging to the crinane-type, were potent inducers of apoptosis in tumour cells. Besides, Extracts of *Narcissus tazetta* var. chinensis, a member Amaryllidaceae family, which induced apoptosis on HL-60 cells contained pseudolycorine, lycorine, tazettine, *etc*. [36]. 

However, some novel research found the induction of apoptosis is not the primary mechanism responsible for antiproliferative activity in some Amaryllidaceae alkaloids such as narciclasine [37], lycorine, amarbellisine, haemanthamine, and haemanthidine [38], and crinine-type alkaloids [39]. 

In present study, oduline and its derivatives 2-hydroxyoduline, 2-hydroxy-6-*O*-methyloduline, and 2-methoxy-6-*O*-methyloduline were found for the first time in *L. aurea*. Their antitumor activity was seldom researched so far. Homolycorine was first isolated from *Lycoris radiata* by Kondo and Tomimura [26], and has been described as non active against tumors to date [40]. 

The stereostructure of hippeastrine has been identified by Uyeo *et al*. in 1954 [41], and it shows selective anticancer activities on several cancer cell lines [42] and induces cancer cell apoptosis [43].

Unlike the extract components [36] and single alkaloids, which belong to the lycorine, crinane, and narciclasine-type alkaloids and induce cancer cell apoptosis, DELA contained lycorerine-type Amaryllidaceae alkaloids, and can also induce murine sarcoma 180 cells apoptosis. Most importantly, DELA showed antitumor activity *in vivo* with low immunotoxicity. 

## 3. Experimental

### 3.1. Materials

The chemicals 3-(4,5-dimethylthiazol-2-yl)-2,5-diphenyltetrazolium bromide (MTT), trypan blue, DMSO, CTX (purity, 98%), penicillin, and streptomycin were all purchased from Sigma Chemical Co. (St. Louis, MO, USA). The RPMI-1640 culture medium and foetal bovine serum (FBS) were purchased from HyClone (Logan, UT, USA). The apoptosis detection kit was purchased from Key Gen Biotech Co. (Nanjing, China). The antibodies to Bax, Bcl-2, procaspase-3, and actin were purchased from Santa Cruz Biotechnology, Inc (Santa Cruz, CA, USA). The PVDF membranes for Western blotting were purchased from Millipore (Billerica, MA, USA). All other chemicals were purchased from local chemical market (Wuhan, China).

Murine sarcoma S180 cells were obtained from the Cancer Research Institute of Hubei Province (Wuhan, China). Male Kunming *albino* mice [SCXK (Hubei) 2008-0005] (20 ± 2 g) were obtained from the Hubei Experimental Animal Center (Wuhan, China). All animals were maintained in accordance to institutional guidelines for the care and use of laboratory animals and the experiments were approved by the Ethics Committee of the Huazhong University of Science and Technology (Wuhan, China).

The LC system consisted of an Agilent 1200 and an injector with a 5 μL loop. The column used was a reversed-phase Eclipse XDB-C18 analytical column (4.6 mm × 150 mm, 5 μm particle size) and was eluted at a flow rate of 0.5 mL/min solvent consisted of 30% MeOH and 70% 10 mM ammonium acetate aqueous solution.

An Agilent LC-MSD-Trap-XCT instrument was operated at a fragmentor voltage of 140 V in the APCI-positive (PI) mode. The gas temperature was 350 °C, the vaporizer was set to 400 °C, nitrogen was supplied as drying gas at 7 L/min, with a nebulizing pressure of 276 kPa. Spectra in the mass range between 100 and 900 were recorded. 

NMR spectra in CDCl_3_ were obtained using An AV-400 nuclear magnetic resonance (NMR) instrument (Brucker Co., Fällanden, Switzerland), operating at 400 MHz for ^1^H and ^13^C. 

### 3.2. Extraction from L. aurea and Isolation of Anticancer Compounds

**Figure 5:**
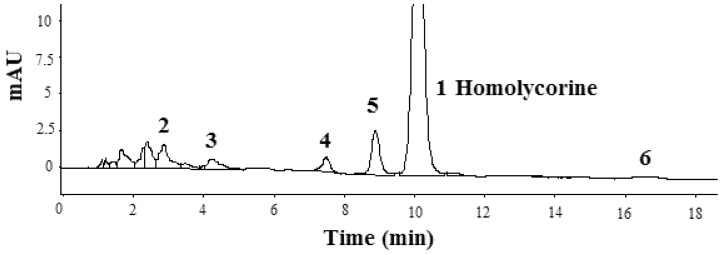
HPLC chromatogram of DELA from *Lycoris aurea*, were recorded at 230 nm.

The bulbs (10.0 kg) were powdered, extracted with 10 times amount of methanol at 70 °C for 3 h, filtered, repeated 3 times, and then concentrated. The concentrate was dissolved in pH 2 hydrochloric acid, and then partitioned successively with petroleum ether and dichloromethane, respectively. The dichloromethane fractions were evaporated on a rotary evaporator under reduced pressure. The dichloromethane residues were subjected to 10 times weight neutral alumina column chromatography eluted with ethyl acetate-petroleum ether (2/8, v/v). The eluent was concentrated and dissolved in methanol and filtered through a Millipore filter (0.45 µm). The filtrate was concentrated and dried to constant weight under vacuum at 50 °C, yielding 0.90 g of extract (the fraction was designated DELA and used to test anticancer activities *in vitro* and *vivo*). HPLC chromatogram of DELA was showed in Figure 5. The extracts were subjected to C_18_ column chromatography eluted with methanol-water (0/100–100/0, v/v) and gave 55 mg of pale yellow crystals of over 95% purity. 

*Homolycorine* (**1**): pale yellow powder. ^1^H-NMR (CDCl_3_, 400 MHz): δ = 7.601 (1H, s, H-8), 7.041 (1H, s, H-11), 5.522 (1H, d, *J* = 2.0 Hz, H-4), 4.781 (1H, t, *J* = 2.0 Hz, H-5a), 3.207 (1H, m, H-2α), 2.736–2.374 (6H, m, H-3, 5, 11b, 11c), 2.291 (1H, dd, *J* = H-2β), 3.992, 3.950 (each 3H, s, OMe), 2.04 (3H, s, NMe). ^13^C-NMR (CDCl_3_, 400 MHz): δ = 56.35 (C-2), 27.86 (C-3), 140.06 (C-3a), 115.79 (C-4), 31.19 (C-5), 77.48 (C-5a), 165.59 (C-7), 117.54 (C-7a), 115.99 (C-8), 145.8 (C-9), 150.98 (C-10), 110.44 (C-11), 136.41 (C-11a), 43.53 (C-11b), 66.65 (C-11c), 56.35, 56.35 (each, OMe), 43.53 (NMe). The above data were identical to those in the literatures [44,45].

### 3.3. Cell Cultures

Murine sarcoma S180 cells were grown in RPMI 1640 culture medium supplemented with 10% FBS (v/v), 100 U/mL penicillin, 100 µg/mL streptomycin at 37 °C under 5% CO_2_.

### 3.4. MTT Assay

The *in vitro* cell death assay following DELA treatments was performed using the MTT method [9]. Briefly, cells were seeded in 96-well plates at a density of 5 × 10^3^ cells per well and cultured with drugs for 48 h. Next, 5 µL MTT (5 mg/mL) was added to each well. The plates were then incubated for an additional 4 h at 37 °C under 5% CO_2_. The culture media was removed and the formazan crystals solubilized with 150 µL DMSO for 15 min. The absorbance at 570 nm was determined using a microplate reader GENios instrument (Mannedorf, Switzerland).

### 3.5. Anticancer Activity *in Vivo*

Murine sarcoma S180 cells were obtained from murine sarcoma S180 tumour-bearing mice, and diluted with RPMI 1640 medium at a ratio of 1:4 (v/v). Then 1 × 10^7^ cells were injected beneath the arm pit into each mouse. All the operations were under the aseptic condition. 50 tumor bearing mice were randomized and divided into five groups at the second day. The model control group, the CTX group, and the high, middle, and low DELA dosage groups were treated with 0.2 mL saline (*i.g.*), 20 mg/kg CTX (*i.p.*), and DELA at 120 mg/kg, 40 mg/kg, and 20 mg/kg (*i.g.*), respectively. 

Treatments were done at a frequency of *i.p.* one time per day for a total of seven consecutive days. DELA-treated groups were more active than model control and CTX-treated groups during treatments. Twenty-four hours after the last dosage, anesthetized with ether, sacrificed by cervical dislocation, and the solid tumours, thymuses and spleens were removed. The inhibition rates of DELA-treated tumour mice were calculated as (C-T)/C × 100%, where T is the average tumour weight (g) of treatment group and C is the average tumour weight (g) of the model control group. The index of thymus was calculated as W_t_/W_m_, where W_t_ is the average thymus weight (mg) of each group and W_m_ is the average mouse body weight (g) of each group. The index of spleen was calculated as W_s_/W_m_, where W_s_ is the average spleen weight (mg) of each group and W_m_ is the average mouse body weights (g) of each group.

### 3.6. Flow Cytometry Assay

Apoptosis-mediated cell death of tumour cells was examined using a double staining method with Annexin V-FITC/PI Apoptosis Detection Kit according to the manufacturer’s instructions. After 24 h of treatment with 0, 10, 20, and 50 µg/mL DELA, the murine sarcoma S180 cells were collected, and washed twice in cold PBS. Then 2 × 10^5^ cells were collected, mixed with 500 µL binding buffer, and stained with 5 µL AnnexinV-FITC and 5 µL PI for 10 min at 25 °C in the dark. Finally, flow cytometry was used to quantify cell death induced by DELA. Data acquisition and analysis were performed by BD FACSCalibur and CellQuest software (Franklin Lakes, NJ, USA).

### 3.7. Western Blotting Assay

Cells were treated with 0, 20, and 50 µg/mL DELA for 12 h, collected and lysed in lysis buffer. Lysates were centrifuged at 12,000 g for 5 min at 4 °C. The concentration of total protein was measured using the BCA assay on an enzyme mark instrument (Mannedorf, Switzerland) at 595 nm. Protein samples were separated on 12% SDS-PAGE gels and transferred onto PVDF membranes. Immune complexes were formed by incubation in antibodies to procaspase-3, Bcl-2, Bax, and actin for 1 h at 37 °C.

### 3.8. Statistical Analysis

All results are presented as means ± SD from triplicate experiments performed in parallel unless otherwise indicated. Statistical analyses were performed using an unpaired, two-tailed Student *t*-test. All comparisons were made relative to the untreated groups and significance of difference is indicated as * *p* < 0.05 and ** *p* < 0.01.

## 4. Conclusions

In summary, our evaluation of *in vivo* and *in vitro* anticancer activity indicated that DELA is a potent inducer of cancer cell apoptosis with low immune toxicity. NMR spectra and LCMS data showed the components of DELA are homolycorine, 2α-hydroxyoduline, oduline, hippeastrine, 2α-hydroxy-6-*O*-methyloduline, and 2α-methoxy-6-*O*-methyloduline.
